# Dissection of Quantitative Blackleg Resistance Reveals Novel Variants of Resistance Gene *Rlm9* in Elite *Brassica napus*

**DOI:** 10.3389/fpls.2021.749491

**Published:** 2021-11-18

**Authors:** Paul Vollrath, Harmeet S. Chawla, Dima Alnajar, Iulian Gabur, HueyTyng Lee, Sven Weber, Lennard Ehrig, Birger Koopmann, Rod J. Snowdon, Christian Obermeier

**Affiliations:** ^1^Department of Plant Breeding, IFZ Research Centre for Biosystems, Land Use and Nutrition, Justus Liebig University Giessen, Giessen, Germany; ^2^Department of Plant Sciences, Crop Development Centre, University of Saskatchewan, Saskatoon, SK, Canada; ^3^Plant Pathology and Crop Protection Division, Department of Crop Sciences, Georg August University of Göttingen, Göttingen, Germany; ^4^Department of Plant Sciences, Faculty of Agriculture, Iasi University of Life Sciences, Iaşi, Romania

**Keywords:** ONT, structural variation, blackleg, *Brassica napus*, long-read sequencing, Rlm9

## Abstract

Blackleg is one of the major fungal diseases in oilseed rape/canola worldwide. Most commercial cultivars carry *R* gene-mediated qualitative resistances that confer a high level of race-specific protection against *Leptosphaeria maculans*, the causal fungus of blackleg disease. However, monogenic resistances of this kind can potentially be rapidly overcome by mutations in the pathogen’s avirulence genes. To counteract pathogen adaptation in this evolutionary arms race, there is a tremendous demand for quantitative background resistance to enhance durability and efficacy of blackleg resistance in oilseed rape. In this study, we characterized genomic regions contributing to quantitative *L. maculans* resistance by genome-wide association studies in a multiparental mapping population derived from six parental elite varieties exhibiting quantitative resistance, which were all crossed to one common susceptible parental elite variety. Resistance was screened using a fungal isolate with no corresponding avirulence (*AvrLm*) to major *R* genes present in the parents of the mapping population. Genome-wide association studies revealed eight significantly associated quantitative trait loci (QTL) on chromosomes A07 and A09, with small effects explaining 3–6% of the phenotypic variance. Unexpectedly, the qualitative blackleg resistance gene *Rlm9* was found to be located within a resistance-associated haploblock on chromosome A07. Furthermore, long-range sequence data spanning this haploblock revealed high levels of single-nucleotide and structural variants within the *Rlm9* coding sequence among the parents of the mapping population. The results suggest that novel variants of *Rlm9* could play a previously unknown role in expression of quantitative disease resistance in oilseed rape.

## Introduction

Oilseed rape/canola (*Brassica napus* L.) is one of the most important vegetable oil crops. As a recent allotetraploid crop, originating from an interspecific hybridization event between its two diploid ancestors *Brassica rapa* (2n = 2x = 20, AA) and *Brassica oleracea* (2n = 2x = 18, CC) (Nagaharu, 1935), *B. napus* (2n = 4x = 38, AACC) carries a highly complex and dynamic genome which is affected by many small-scale and large-scale structural variations (Parkin et al., 1995; Chalhoub et al., 2014; Stein et al., 2017; Hurgobin et al., 2018; Chawla et al., 2020). Many studies revealed high frequencies of homoeologous exchanges between the highly similar A and C subgenomes (Szadkowski et al., 2010; Chalhoub et al., 2014; Samans et al., 2017).

The hemibiotrophic fungal pathogen *Leptosphaeria maculans* (Desm.) Ces. & de Not. [anamorph: *Phoma lingam* (Tode ex. Fr.) Desm.] causes stem canker in *B. napus*. This disease, also known as blackleg, is a major problem in almost all oilseed rape and canola-growing regions around the globe. Substantial yield losses have been reported in Australia, North America and several European countries (Fitt et al., 2006). The primary infection of winter oilseed rape takes place in autumn via airborne ascospores. Additionally, secondary infections are likely through pycnidiospores formed within the asexual pycnidia. The spores penetrate the host tissue via stomata or wounds and colonize intercellular spaces of the mesophyll. From there, the fungus starts its symptomless, biotrophic growth systematically through the petiole into the stem. Here the pathogen kills the cells, leading to girdling and rotting of the stem base. As a result, the plant tends to ripen prematurely and severe infections can lead to serious lodging and death (West et al., 2001). Resistance breeding is the most sustainable and effective method to counteract *L. maculans*. Resistance of *B. napus* against *L. maculans* is often divided into two classes of resistance mechanisms. A distinction is made between race-specific, qualitative resistance determined by major genes, and non-race-specific quantitative resistance provided by numerous minor effect genes. Qualitative resistance against *L. maculans*, considered as complete resistance, has been investigated in considerable detail and used extensively in commercial breeding programs of *B. napus*, due to the high efficacy and convenient assessment at the cotyledon stage (Rimmer and van den Berg, 1992; Balesdent et al., 2005; Delourme et al., 2006; Elliott et al., 2016). However, it has been observed that rapid adaptation of the pathogen populations can overcome *R* gene-mediated resistance in the field within a few seasons (Rouxel et al., 2003; Sprague et al., 2006; Brun et al., 2010; Zhang et al., 2016; van de Wouw et al., 2017). Thus, commercial breeders place special focus on quantitative disease resistance. Quantitative resistance is influenced by multiple genes, and the incomplete nature of the resistance decreases the selection pressure on the pathogen population and consequently increases the durability of the resistance (St Clair, 2010; Delourme et al., 2014). In contrast to qualitative resistance, the assessment of quantitative resistance is more challenging as it is mainly expressed at adult plant stages and highly influenced by environmental conditions (Fitt et al., 2006; Huang et al., 2009). A main target of breeding is therefore the combination of highly effective *R* gene-mediated resistance with a broad and durable quantitative resistance (Brun et al., 2010; Pilet-Nayel et al., 2017). However, this clear distinction between the two types of resistance has recently been questioned in several studies (Thomma et al., 2011; Delplace et al., 2020). Also, in some cases the expression of partial resistance in adult plants imparted by major resistance genes has been demonstrated (Chantret et al., 1999; Raman et al., 2018). The complexity of the *B. napus*–*L. maculans* pathosystem and possible corresponding genes often makes the assessment of quantitative resistance difficult. Therefore, the objective of the present study was to identify genomic regions involved in quantitative blackleg resistance by excluding the possibility of effective *R* gene-*Avr* gene interactions. A multiparental mapping population was tested under controlled greenhouse conditions with a selected highly virulent fungal isolate. In addition, whole-genome long-read re-sequencing using Oxford Nanopore Technology (ONT) was conducted to reveal the implications of single nucleotide variants (SNV) and structural genome variations (SV) on an agronomical highly important trait within the narrow genepool of European elite winter oilseed rape. Recently, Chawla et al. (2020) demonstrated a previously unknown extent of genome-wide, small to medium-sized SV events within *B. napus* genes using ONT long-read sequencing technology. In contrast, SNV have to date been largely ignored in quantitative trait analysis of important crop traits, due to the difficulty of assaying SV on a genome-wide scale in complex crop genomes. In the past few years, however, rapidly decreasing costs and increasing accuracy of long-read sequencing from the ONT or Pacific Biosciences platforms has opened the way to include genome-wide SV data from long-read sequences in QTL analysis and interpretation. Here we successfully called SNV in ONT data from the seven elite winter oilseed rape parents of the multiparental mapping population, enabling us to associate genome-wide SV with single nucleotide polymorphism (SNP) haplotypes carrying blackleg resistance QTL.

## Materials and Methods

### Plant Material

A *B. napus* multiparental population comprising 354 double haploid (DH) lines derived from seven European elite winter oilseed rape varieties was tested for quantitative blackleg resistance in multiple greenhouse screenings. The mapping population consisted of six subfamilies derived from crosses of the elite parent “Lorenz” to six elite founder lines (“Adriana,” “Alpaga,” “DK Cabernet,” “Galileo,” “King 10,” and the DH line “JN”). Each of the subfamilies comprised 60 DH lines except for the cross Lorenz × Galileo, which comprised 54 DH lines. The common parent Lorenz was previously classified as highly susceptible to blackleg disease, whereas the other six founder lines were all known to carry quantitative blackleg resistance (unpublished data, breeding companies). The German breeding companies NPZ Innovation GmbH (Holtsee, Germany), Syngenta Seeds GmbH (Bad Salzuflen, Germany), and KWS SAAT SE & Co. KGaA (Einbeck, Germany) produced and provided the DH families. In parallel, another panel of 256 diverse winter oilseed rape inbred lines was tested for blackleg resistance in a 2-year field trial with one plot per field. These accessions were part of the ERANET-ASSYST *B. napus* diversity set, previously described by Bus et al. (2011).

### Resistance Screenings and Data Analysis

The multiparental mapping population was screened for adult-plant blackleg resistance under controlled conditions in the greenhouse of Georg August University of Göttingen in 11 independent screening experiments, each individual screening included all 354 genotypes with two plant individuals (replicates) per genotype in a completely randomized design. In total 22 plant individuals were tested per genotype across 11 screening experiments (22 replicates per genotype). Manual infection was carried out at developmental stage BBCH 13–14 (3–4 true leaves, leaf pairs, or whorls unfolded) (Lancashire et al., 1991). *L. maculans* was propagated on oatmeal agar medium 2 weeks before infection. A mycelial agar plug was then placed at the stem base, slightly above the axil of the first true leaf, after wounding of the infection site using a needle. Subsequently, plants were grown under foil tunnels for 72 h to ensure appropriate humidity and temperature for a successful infection. At 49 days post infection (dpi), a cross section was cut at the stem base to estimate the length (L), girdling (G), and penetration depth (P) of the blackleg lesions. L was measured in mm whereas G and P were visually scored as percentage of the total circumference and diameter, respectively. Next, these scores were converted into individual 0–9 scales for each score. Using L, G, and P scoring values at 49 dpi, the Volume of Diseased Tissue (VDT) value was calculated using a formula modified from Kutcher et al. (1993):


$$V\,D\,T=\left(1-{\left(\frac{1-P}{9}\right)}^{2}\right)*\frac{G}{9}*L$$

Also, single screening means of the two replicates per screening, adjusted means across all the eleven screenings, were calculated using the R packages lmerTest version 3.1-2 (Kuznetsova et al., 2017) and lsmeans version 2.30-0 (Lenth, 2016). This approach allowed the assessment of QTL stability across different screenings.

The diversity set was grown in 2016/2017 and 2017/2018 in field trials in Rauischholzhausen, Germany. It was grown under normal farming practices with no use of fungicides. The fields were chosen based on close crop rotation and known high natural blackleg infection pressure. Single plots per genotype were sown and analyzed in a randomized complete block design, with plot sizes of 12.5 m^2^ (10 m × 1.25 m). At developmental stage BBCH 83–85 (30–50% of pods ripe, seeds black, and hard), 20 plants from the middle row of each plot were uprooted and cut at the stem base. Visual scores from 1 to 6 for blackleg infestation at the resulting cross section were used to calculate the G2 index for blackleg adult plant stem infection (Pilet et al., 1998; Aubertot et al., 2004) using the formula:


G⁢2⁢i⁢n⁢d⁢e⁢x=[(N⁢1⁢x⁢0)+(N⁢2⁢x⁢1)+(N⁢3⁢x⁢3)+(N⁢4⁢x⁢5)+(N⁢5⁢x⁢7)+(N⁢6⁢x⁢9)]/N⁢t,

where N1, N2, N3, N4, N5, and N6 are the number of stems with scores 1, 2, 3, 4, 5, and 6, respectively, and Nt is the total number of stems assessed.

### Characterization of Fungal Isolates

Leaf samples with characteristic lesions of *L. maculans* were collected in the field trials and dried. Leave segments were incubated in humid chambers to allow spore release from pycnidia. Single pycnidium isolates were prepared by plating spores on SNA medium amended with 200 ppm streptomycin for 6 days. Petri dishes were incubated under UV light at 20°C. Subsequently, a mycelial plug was transferred to V8 medium supplemented with 200 ppm streptomycin and incubated for 2 weeks under the same conditions. Spore suspensions were prepared by adjustment to 1 × 10^7^ spores/ml using a hemocytometer. To characterize *L. maculans* isolates, cotyledon tests were applied using a differential set of *B. napus* harboring major resistance genes (Supplementary Table 3; Balesdent et al., 2002, 2006; Marcroft et al., 2012). Shortly, 10 μl spore suspension was applied on each lobe of cotyledon after injuring it with a needle. Eight replications were used. Symptoms were evaluated 14 days post inoculation according to the IMASCORE rating scale, where class 1 shows typical hypersensitive reactions and class 6 reflects tissue collapse with sporulation. Classes 1–3 were considered as resistance reactions, while classes 4–6 were noted as susceptible ones (Balesdent et al., 2001).

### Single Nucleotide Polymorphism Genotyping and Analysis of Linkage Disequilibrium

All the investigated *B. napus* accessions were genotyped using the *Brassica* 60k Illumina Infinium™ SNP array. The *B. napus* reference genome assembly Darmor-*bzh* version 10 (Rousseau-Gueutin et al., 2020) was used to anchor 34,079 markers uniquely to a single position of the genome (Supplementary Table 1). Markers mapping unspecifically to multiple positions were excluded from further analyses. Finally, single hits were filtered for a cut-off *e*-value of 1e^––15^. Heterozygote SNP calls were considered as missing data since we should not expect heterozygote calls for DH or inbred lines, hence it can be assumed that these calls are mostly due to technical artifacts. Genome-wide linkage disequilibrium (LD) was calculated using the R package SelectionTools version 19.4^1^. Prior to LD analysis, markers were filtered for minor allele frequency (MAF) ≥0.05 and a maximum of 10% missing data per marker and DH line. A tolerance threshold of *r*^2^ > 0.4 was set to assign markers in strong LD to respective LD blocks.

### Genome-Wide Association Studies

Genome-wide association studies were conducted using the R package GenABEL version 1.8-0 (Aulchenko et al., 2007). Markers for the multiparental population were filtered as described above for LD analysis. This approach led to a set of 17,869 polymorphic and unique markers, including 16,400 SNP markers and 1,469 Single Nucleotide absence Polymorphism (SNaP) markers called as described by Gabur et al. (2018). The linear mixed model was adjusted for population structure by consideration of identity-by-state estimates and the first two principal components (PC) as covariates. To reduce false positive rates, LD blocks were determined as suggestive QTL when a minimum of two markers per block showed trait associations in at least two individual greenhouse screening rounds. A LOD score of −log10 (*p*-value) ≥3.0 was applied as threshold for suggestive marker-trait associations. Finally, QTL were determined after correction for false discovery rate (FDR ≤ 0.1) when performing GWAS with the adjusted means across all greenhouse screening rounds.

In order to validate QTL discovered in the multiparental population using the greenhouse data, we also performed an independent GWAS in the diversity panel using phenotypic data from the field trials. Similar filtering steps led to 23,603 polymorphic SNP markers. Here, LD-based QTL were defined by considering kinship and PC and applying a LOD score of −log10 (*p*-value) ≥3.0 as an arbitrary threshold for putative marker-trait associations.

### Functional Annotation of Darmor-*bzh* Genes

Functional annotation data for Darmor-*bzh* v4.1 produced by Gabur et al. (2020) were used together with genome-wide functional annotation of Darmor-*bzh* v10 performed using the ‘‘Automatic assignment of Human Readable Descriptions’’ (AHRD)^2^ package (Supplementary Table 2). AHRD obtains the functional annotations for gene models by blasting them to various publicly available protein databases such as Swiss-Prot, TAIR or trEMBL. Two hundred best scoring blast results (based on *e*-value) were chosen from each of the above-mentioned databases. Description for all the resulting blast hits was then assigned a score using a multi-step approach. In the first step every description line was subjected to a couple of regular expression filters, removing descriptions such as “Whole genome shotgun sequence” and other vague terms like “OS = Arabidopsis thaliana.” In the subsequent step the description lines were broken down into single tokens. These tokens were then pushed through a blacklist filter, thereby discarding all the tokens present in the blacklist. Every token was then assigned an overlap score based on the bit score, the database score, and the overlap score of the blast match. In the last step the token score was divided by a correction factor to remove any bias toward longer or shorter description lines. For the exact database and software versions please refer to the “Darmor10_input_go_prediction.yaml” file in the Supplementary Material.

### Whole Genome Long-Read Resequencing and Variant Calling

Long-read sequencing was performed for all the seven parental lines of the mapping population using Oxford Nanopore Technologies (ONT). DNA extraction was conducted as described in Chawla et al. (2020). In addition, DNA size selection was performed using the Circulomics Short Read Eliminator Kit (Circulomics Inc.). The recommended kit LSK-109 from ONT was used for DNA library preparation. Long-read sequencing was performed using the MinION device from ONT. Subsequently, basecalling was executed with Guppy version 4.0.14 and reads were aligned to the *B. napus* reference assembly Darmor-*bzh* v10 using the long-read mapper NGMLR version 0.2.7 (Sedlazeck et al., 2018). BAM files were created using samtools version 1.9 (Li et al., 2009). For genome-wide SV detection the variant caller Sniffles version 1.0.12 was used with default settings (Sedlazeck et al., 2018). Deletions and insertions with a minimum size of 30 bp were classified as SV. In addition, SNV calling was conducted using the deep neural network based variant caller Clair version 2 (Luo et al., 2020). The Clair module callVarBam, with a model for ONT data, was used to call SNV from BAM files. To reduce false positive calls, SNV calls were filtered for a minimum quality using a shell script kindly provided by Fritz Sedlazeck and Medhat Mahmoud, Baylor College of Medicine, Human Genome Sequencing Center, Houston, TX, United States. The script calculates the most appropriate cut-off value for SNV quality filtering according to the recommendations of the authors of Clair. Luo et al. (2020) observed that quality scores of variants derived from ONT data are usually bimodally distributed, so that high-quality base calls can be extracted by setting a quality cut-off at a value corresponding to the bottom of the valley between the two peaks plus 50. In addition to quality filtering, a stringent filtering for only homozygous calls and a minimum allele frequency (AF) of 0.5 for the variant were applied. Then, the files of the single genotypes were merged using bcftools version 1.10.2 and were subsequently used as an input to invoke the force-calling parameter of Clair. Next, all samples were run again to force-call the SNV provided within the merged file. This again was followed by filtering for quality, homozygous calls and a minimum AF of 0.1. A lower threshold for AF was chosen after force-calling, since previous SNV calling already indicates the presence of these variants. Subsequently, CLC Genomics Workbench (v9.0, QIAGEN Digital Insights, Aarhus, Denmark) was used to align the sequences of the parental lines and to predict the impacts of amino acid changes caused by SNV.

### Single Nucleotide Variants Validation

Due to the high number of SNV, the sequence of the gene A07p27010.1_BnaDAR was selected to validate the SNV calling method. For this purpose, sets of primers were designed to amplify the selected region and Sanger Sequencing was performed in the mapping parents. Primers were designed using the online tool Primer3Plus (Untergasser et al., 2007).

## Results

### Identification of a *L. maculans* Isolate Without *R* Gene Interaction for Use in Quantitative Resistance Screening

From the 644 *L. maculans* isolates collected in Northern Germany and characterized by Winter and Koopmann (2016), one isolate was selected (isolate 1.4.1.15). This isolate has been shown in cotyledon test with a *B. napus* differential set to harbor virulence alleles against *R* genes *Rlm1*, *Rlm2*, *Rlm4*, *Rlm7*, *Rlm9, LepR2*, and *LepR3*. The isolate was tested on the cotyledons of the seven elite oilseed rape parents of the multiparental mapping population. It showed a susceptible interaction in all cotyledon tests indicating that none of the parents exhibit any qualitative major resistance against this fungal isolate based on plant *R* gene and fungal avirulence gene interaction (Supplementary Table 3). Thus, this virulent isolate 1.4.1.15 recovered from a field in Peine (Germany) in 2013 was used subsequently for the greenhouse screenings for quantitative resistance, in order to avoid interaction of major monogenic resistance genes with fungal avirulence genes that could putatively mask the effects of minor quantitative resistance loci in the mapping population.

### European Elite *B. napus* Accessions Show Genetic Variation for Quantitative Blackleg Resistance

Mixed linear models (MLM) demonstrated significant genotypic variation within the multiparental population (*p* < 0.001). VDT values from greenhouse screenings showed a normal distribution within the six subfamilies as well as in the entire mapping population, confirming the quantitative inheritance of blackleg resistance in this population (Supplementary Figure 1). LD analysis resulted in an average number of 33 LD blocks per chromosome. Single greenhouse screening rounds using mean VDT values from 2 replicates per genotype revealed together up to 326 marker-trait associations in GWAS. GWAS using mean VDT values composed of 22 replicates for each genotype merged from a total number of 11 screening rounds identified 84 significant marker-trait associations (Supplementary Table 4). After FDR correction a total of eight QTL regions were identified (Table 1), seven on chromosome A09 and one on chromosome A07. No QTL were detected on C-subgenome chromosomes. As expected for quantitative resistance, phenotypic variation explained by individual SNP markers was low and was ranging from 3.2 to 5.6% (Table 1). QTL stacking in the multiparental population revealed that the allele combination present in the common parent Lorenz led to the highest susceptibility compared to alleles from the other parental lines. This confirmed the initial assumption and the choice of Lorenz as the susceptible common parent. Almost all resistance alleles were derived from the six founder lines, whereas only one resistance allele was derived from the common parent Lorenz. The ideal allele combination of the eight QTL resulted in an estimated effect on resistance of 35.5% (−2.06 on the VDT scale). This beneficial combination was already present in the two founder lines DK Cabernet and JN (Supplementary Table 5).

**TABLE 1:** Blackleg resistance QTL (VDT trait) identified in a *B. napus* multiparental elite mapping population in 11 independent greenhouse screening rounds (*n* = 354) and overlapping resistance QTL (G2 or stem lesion) in the ERANET-ASSYST diversity set in two field trials (*n* = 256).

		Multiparental elite mapping population					ERANET-ASSYST diversity set				
QTL ID	Chromo-some	Start-end position of LD block/QTL	Size of QTL	LOD#	*R*^2^#	Detection in screening rounds*	Start-end position of LD block/QTL	Size of QTL	LOD#	*R*^2^#	Detection in field trial
A07.b304	A07	20,107,508–20,995,393	888 kb	3.35	3.40%	T, 3, 6, 7, 9, 10	20,079,904–20,378,231	298 kb	3.57	7.79%	2017/18
A09.b366	A09	13,704,742–14,702,671	998 kb	5.17	5.48%	T, 1, 3, 7	12,981,315–14,141,992	1,160 kb	3.46	6.82%	2016/17
A09.b369	A09	15,852,358–16,237,668	385 kb	5.22	5.53%	T, 1, 3, 7	ns	ns	ns	ns	–
A09.b370	A09	16,917,123–16,939,597	22 kb	3.17	3.19%	T, 1, 3	ns	ns	ns	ns	–
A09.b374	A09	18,825,078–19,494,241	669 kb	5.07	5.36%	T, 2, 3, 11	ns	ns	ns	ns	–
A09.b380	A09	33,761,934–33,966,136	204 kb	5.02	5.32%	T, 1, 2, 3, 11	ns	ns	ns	ns	–
A09.b381	A09	34,149,762–38,832,384	4,683 kb	3.50	3.58%	T, 1, 3	ns	ns	ns	ns	–
A09.b382	A09	38,844,647–41,182,649	2,338 kb	3.88	4.09%	T, 1, 3	41,110,836–42,454,513	1,343 kb	3.07	5.53%	2016/17

#LOD and R^2^ values for peak markers; *T = data from 11 screening rounds combined (adjusted mean values), numbers indicate different screening rounds with 2 plant individuals per genotype; ns, not significant.

### Candidate Gene Analysis for Genes Involved in Quantitative Resistance

Based on two approaches together we identified 128 genes across the eight QTL regions that are associated to defense response and/or resistance in at least one reference genome (Supplementary Table 6). The QTL on chromosome A07 (haploblock A07.b304) was detected in the highest number of individual screening rounds (Table 1). The haploblock had a size of 888 kb in the investigated multiparental mapping population and contained 53 SNP markers and 193 genes in Darmor-*bzh* v10 (Supplementary Table 7). However, some genes in the interval revealed no annotation in Darmor-*bzh* v4.1 because no homologous genes exist between Darmor-*bzh* v4.1 and v10 or because Blast2GO revealed no annotation for the Darmor-*bzh* v4.1 genes (60 of 193). Thus, the protein sequences of the 193 *B. napus* Darmor-*bzh* v10 genes from the QTL interval were additionally aligned to the Arabidopsis reference genome Araport11 (Cheng et al., 2017) and literature links were evaluated exhibiting some additional, more detailed functional annotations. Out of 193 genes, 17 were associated to defense response and/or resistance (Supplementary Table 7). In particular, we found substantiated evidence in the literature that three of these 17 genes have a function related to fungal plant resistance in *A. thaliana* and *B. napus* in interaction with common *B. napus* fungal pathogens (A07p26890.1_BnaDAR, A07p28430.1_BnaDAR, A07p27010.1_BnaDAR). Most interestingly, the gene A07p27010.1_BnaDAR has been shown to be the major resistance gene *Rlm9*, which imparts qualitative resistance against *L. maculans* in *B. napus* (Larkan et al., 2020). To evaluate for the presence of resistance gene analogs (RGAs) in the QTL region of A07.b304 and their polymorphisms between the parents of the multiparental mapping population, we also located the QTL interval for Darmor-*bzh* versions 4.1 (Chalhoub et al., 2014) and 8.1 (Bayer et al., 2017) based on the flanking SNP markers (Supplementary Table 8). However, two out of five putative RGAs identified in this interval showed no polymorphism and none of them had been annotated to be involved in plant resistance.

### Identification of Major Resistance Gene *Rlm9* as Candidate Gene for Quantitative Blackleg Resistance

**FIGURE 1:**
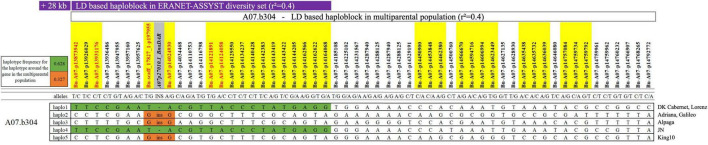
LD based haploblock A07.b304 (*r*^2^ = 0.4) containing 54 single nucleotide polymorphism (SNP) markers and *Rlm9* (A07p27010.1_BnaDAR). Green color indicates the two haplotypes with 24 identical SNP markers between the three parental lines Lorenz, DK Cabernet and JN. Haplotypes 2, 3, and 5 are harboring the 6 kb insertion in the sequence of *Rlm9* (orange). Purple bar highlights the overlap with the LD based QTL identified in the diversity set in field trials. Gray highlight indicates Rlm9 gene, yellow highlight indicates SNP markers identified in one individual greenhouse screening round, red color indicates SNP markers identified in at least two individual greenhouse screening rounds.

The detected quantitative resistance region has been mapped before for the qualitative resistance gene *Rlm9* in young seedlings (Larkan et al., 2016b; Raman H. et al., 2020). However, testing of virulence complexity proved that the isolate used for greenhouse screening for quantitative resistance in our experiments shows no gene-for-gene interaction with *Rlm1*, *Rlm2*, *Rlm4*, *Rlm7*, *Rlm9, LepR2*, and *LepR3* (see above). On the other hand, variant calling using ONT long-read sequencing data revealed that the sequence diversity in the gene A07p27010.1_BnaDAR (*Rlm9*) was considerably higher than in all other genes within the QTL interval and all putative resistance genes in any of the other identified QTL (Supplementary Table 7). Within the QTL interval, the peak SNP marker lies just 35 kb away from the *Rlm9* gene. Within the sequence of *Rlm9*, we found 142 polymorphic SNV calls and a 6 kb insertion in four of the seven parental lines (Adriana, Alpaga, Galileo, and King 10). Parental lines Lorenz (susceptible), DK Cabernet and JN displayed an identical haplotype, comprising 24 SNPs in LD to the peak SNP marker, whereas the resistant genotypes harboring the insertion within the sequence of *Rlm9* (Adriana, Alpaga, Galileo, King 10) showed a deviating haplotype (Figure 1). PCR and Sanger sequencing proved both, the authenticity of the 6 kb insertion and the correctness of the 142 intragenic SNV calls in A07p27010.1_BnaDAR. Each of the 142 SNV calls from ONT data was confirmed to be correct, whereby the Sanger sequencing also revealed a further 60 SNV within this particular gene. These 60 false negatives can be explained by the strict filtering process applied to eliminate false positive calls in the SNV calling approach. Nevertheless, the results indicate that SNV calling from “noisy” long reads produced with ONT provides reliable variant calls for genetic analysis.

**FIGURE 2:**
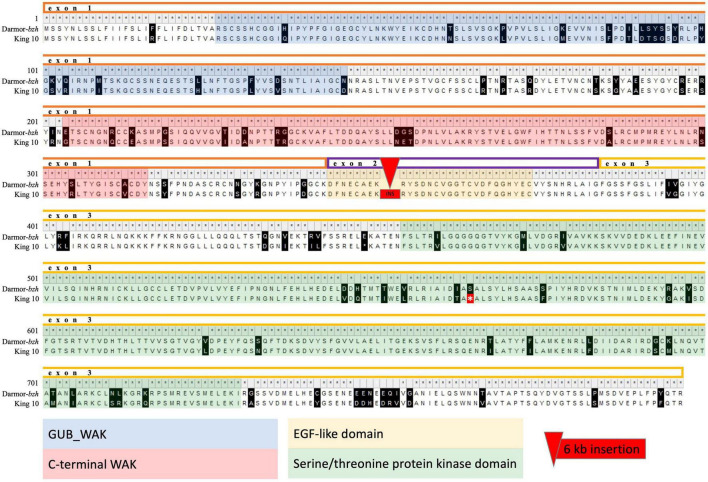
Alignment of the protein sequence of King 10 against the reference Darmor-*bzh* v10. King 10 is representative for the four parental lines harboring a 6 kb insertion and 202 SNV leading to 92 non-synonymous amino acid changes. Different colors indicate exons and conserved domains. The asterisk indicates a stop codon.

*Rlm9* codes for a wall-associated kinase like (WAKL) protein. A search for motifs using the Conserved Domain Search tool implemented in NCBI along with the Pfam database revealed three conserved domains, two within exon 1 and one within exon 3. These comprise an extracellular galacturonan-binding domain (GUB_WAK), a C-terminal wall-associated kinase (WAK) and an intracellular Serine/Threonine protein kinase domain (Ser/Thr_kinase). In addition, an EGF-like domain is located in exon 2 (Larkan et al., 2020). Based on the Sanger sequencing we found that three of the seven parental genotypes (Lorenz, DK Cabernet, JN) carry an identical *Rlm9* allele to Darmor-*bzh*, whereas the other four harbor a 6 kb insertion within the second exon along with 202 SNV throughout the entire gene (Adriana, Alpaga, Galileo, King 10). These SNV caused in total 92 non-synonymous amino acid changes. We observed 19 amino acid changes within the GUB_WAK domain (84.2% identity to Darmor-*bzh*), 15 amino acid changes within the WAK domain (86.7% identity to Darmor-*bzh*) and 23 amino acid changes as well as a stop codon within the Ser/Thr_kinase domain (91.3% identity to Darmor-*bzh*). The EGF-like domain in exon 2 was disrupted by the 6 kb insertion in genotypes Adriana, Alpaga, Galileo, and King 10 (Figure 2).

### Candidate Genes in Co-localizing Quantitative Trait Loci From Greenhouse and Field Trials

The QTL regions and candidate genes where initially identified in the elite population under controlled conditions excluding *R* gene interaction. To putatively support these candidate genes, we also identified QTL for adult plant resistance under field conditions in a diversity set. The field trials using the ERANET-ASSYST diversity set relied on natural infection. Characterization of field isolates sampled at the field site in Rauischholzhausen (Germany) revealed fungal isolates harboring the avirulence alleles *AvrLm3* or *AvrLm7* in combination with virulence alleles *avrLm1, avrLm2, avrLm4*, and *avrLm9*. This situation represents the typical German field situation as reported previously (Winter and Koopmann, 2016). This implies that monogenic *Rlm3* and *Rlm7*, also located on chromosome A07 (Larkan et al., 2016b; Raman R. et al., 2020) and potentially harbored by some of the genotypes of the diversity set, could mask quantitative resistance associated with the investigated genomic region on chromosome A07. However, monogenic *Rlm9* effects should not mask quantitative resistance effects associated with *Rlm9* in this field situation. Three of the eight QTL found in the analysis with the multiparental mapping population were also found in field trials for blackleg stem lesions in the ERANET-ASSYST diversity set (QTL A07.b304, A09.b366 and A09.b382, Table 1 and Supplementary Table 9). The LD blocks for these three QTL partly overlap in both populations. Assuming that the same genes are involved in quantitative resistance expression in both winter oilseed rape populations, the overlap in the QTL interval might be useful to narrow the search for potential candidate genes. By considering overlapping QTL regions as high-confidence QTL intervals, we reduced the areas of interest from 888 kb to 271 kb, 998 kb to 473 kb, and 2,338 kb to 72 kb, respectively. In total, these three intervals contained 133 genes (68, 54, and 11). The number of candidate genes was reduced from 68 to 5, 54 to 2, and 11 to 1 for the three co-localizing QTL intervals for QTL A07.b304, A09.b366, and A09.b382 by filtering for GO terms associated to defense response and/or resistance (Table 2 and Supplementary Table 10). *Rlm9* is localized in the overlapping QTL region of both populations, the multiparental mapping population and the diversity set tested under controlled conditions in the greenhouse and in the field, respectively.

**TABLE 2:** Genes within the *L. maculans* resistance QTL of the multiparental population overlapping with QTL of the diversity set based on GO terms that can be associated with plant resistance and numbers of genomic variants in the multiparental population.

Gene ID	QTL ID	Range of overlap in kb	Gene annotation	Structural variants (SV)	No. of single nucleotide variant (SNV) calls
A07p26790.1_BnaDAR	A07.b304	271 kb	Nuclear pore complex protein NUP96	–	1
A07p26870.1_BnaDAR	A07.b304	271 kb	Cinnamoyl CoA reductase	–	18
A07p26890.1_BnaDAR	A07.b304	271 kb	Transcription factor WRKY	–	17
A07p27010.1_BnaDAR	A07.b304	271 kb	Wall-associated receptor kinase-like 10	6 kb insertion	142
A07p27130.1_BnaDAR	A07.b304	271 kb	ALA-interacting subunit	–	–
A09p21820.1_BnaDAR	A09.b366	473 kb	Protein ENHANCED DISEASE RESISTANCE 2-like	–	96
A09p21880.1_BnaDAR	A09.b366	473 kb	Heat shock transcription factor	–	8
A09p44190.1_BnaDAR	A09.b382	72 kb	UPF0183 protein	–	–

## Discussion

Quantitative resistance against *L. maculans* exhibiting minor effects in *B. napus* is difficult to detect as it is frequently masked by qualitative resistances exhibiting major effects controlled by race-specific *R* genes in the host and pathogen. Pathogen-associated molecular patterns (PAMPs) are considered to initiate a broad-spectrum resistance against a pathogen species, termed PAMP-triggered immunity (PTI), whereas race-specific pathogen effectors contribute to pathogen virulence and can induce effector-triggered immunity (ETI) in the plant host on a gene-for-gene interaction model, also called qualitative resistance (Jones and Dangl, 2006). However, in the last decade it has become clear that this strict distinction between PTI and ETI might need reconsideration (Thomma et al., 2011). In addition, the extent to which PTI is associated with quantitative resistance expression also remains to be clarified (Delplace et al., 2020). ETI and PTI are both mainly involved in pathogen perception and signaling, whereas quantitative resistance goes beyond that and is controlled by numerous genes with diversified functions (Corwin and Kliebenstein, 2017). Qualitative and quantitative resistance cannot normally be clearly distinguished in field studies. The complexity of the fungal population in the field, harboring different avirulence genes, and the genetic composition of the plant population, harboring unknown minor and major resistance allele combinations, generally cause difficulties in association studies of quantitative resistance under field conditions. Thus, our strategy in this study was to identify and use a fungal *L. maculans* isolate that has no qualitative resistance *R* gene interaction with the parental oilseed rape cultivars of our multiparental population, and to use this isolate to map quantitative minor effect QTL by GWAS under controlled conditions.

Using this strategy, we identified a normal distribution of disease, indicating the exclusively quantitative inheritance of blackleg resistance in the mapping population. The absence of effective gene-for-gene interactions that can usually be observed in the *B. napus–L. maculans* pathosystem allowed us to identify genomic regions explaining only a small portion of phenotypic variation (<6%). In accordance with a previous study, we also observed high QTL-by-environment interactions, which is common for quantitatively inherited disease resistance even under controlled greenhouse conditions (Obermeier et al., 2013) and is even more common due to varying disease pressure throughout field trials (Huang et al., 2016; Larkan et al., 2016a; Kumar et al., 2018; Raman et al., 2018).

In most studies mapping quantitative blackleg resistance, different methods for disease scoring of cross sections at the crown or scoring of survival rate were used to estimate the disease severity (Huang et al., 2016; Larkan et al., 2016a; Gabur et al., 2018; Kumar et al., 2018; Raman et al., 2018; Raman R. et al., 2020). In the field trials of the present study, G2 index scoring of cross sections at the crown was applied due to the immense workload that needs to be achieved within a short period of time. However, the G2 index depends strongly on precise cuts to accurately assess the cross sections. This phenotyping method is time-saving compared to VDT scoring and provides useful and informative data from field evaluations of blackleg disease. In addition, to gain more detailed insights into the growth of the fungus, the VDT scoring method of Kutcher et al. (1993) was used for the assessments in the greenhouse trials. The high overlap of QTL results with previous studies underpins the relevance of the applied phenotyping method and further reveals that some of the identified QTL regions are crucial within international germplasm (Jestin et al., 2015; Raman et al., 2016, 2018; Kumar et al., 2018; Fikere et al., 2020; Raman H. et al., 2020; Raman R. et al., 2020; Supplementary Table 11).

We mapped quantitative resistance on chromosomes A09 and A07 in the multiparental population in the greenhouse. All QTL explained less than 5% of the phenotypic variation suggesting that genomic selection approaches are more suitable than marker-assisted selection for breeding toward quantitative *L. maculans* resistance in oilseed rape. Some of the QTL regions identified in the present study on chromosome A09 and A07 overlapped with previously described QTL (Supplementary Table 11). However, chromosome A07, in contrast to A09, is mainly known to harbor qualitative resistance genes providing race-specific resistances (Delourme et al., 2006). Although we ensured the specific assessment of quantitative resistance by selecting a fungal isolate with no gene-for-gene interaction with the mapping population in our greenhouse trials, to our surprise a QTL explaining less than 5% of the phenotypic variance was identified in a genome region on chromosome A07 known to harbor a cluster of *R* genes (*Rlm3*, *Rlm4*, *Rlm7, and Rlm9*) involved in qualitative resistance expression. This suggests that this genome region is either a genomic hot spot where genes involved in qualitative as well as quantitative resistance are tightly clustered (linkage) or that some genes known to be involved in major qualitative resistance can also impart quantitative effects on resistance. Although it is possible that linkage exists between *R* gene clusters which are non-functional at the cotyledon stage in our population and genes involved explicitly in quantitative resistance expression in the LD block on chromosome A07, no clear candidate genes could be identified by GO analysis or polymorphism detection between the parental genotypes. The most striking polymorphism was detected in the well described major resistance gene *Rlm9*, which could suggest that this gene is also involved in adult plant resistance expression in the investigated multiparental population. In addition, *Rlm9* co-localized with an overlapping QTL region in a diversity set for field resistance in adult plants subjected to infection by an *L. maculans* population with an avirulent *AvrRlm9* gene composition. This observation supports the involvement of this candidate gene in quantitative resistance expression in these environments and populations.

This result is in accordance with previous reports that some major *Rlm*/*LepR* genes also have quantitative effects on adult plant resistance. For example, the fungal *AvrLmS-Lep2* gene-for-gene interaction with a *B. napus R* gene, *LepR2*, shows a qualitative intermediate resistance response at the cotyledon stage and partial resistance at the adult plant stage (Long et al., 2011; Dandena et al., 2019; Neik et al., 2020, preprint). The hypothesis that *Rlm9* is involved in quantitative resistance is also supported by Raman et al. (2018), who reported a quantitative resistance effect of the location harboring *Rlm9* in the greenhouse on adult plants, using a plant population segregating for *Rlm9* and an *L. maculans* isolate carrying a corresponding functional avirulence gene *AvrLm5-9* allele. That result also suggested that *Rlm9*-mediated resistance was expressed at the adult plant stage. The concept of *R* genes mediating quantitative resistance has been previously suggested and discussed in other studies that made similar observations (Chantret et al., 1999; Raman et al., 2018). A possible weak, constitutive expression of *R* genes at adult plant stage may lead to partial resistance with only minor effects. Our results suggest that further studies investigating *R* gene expression at adult plant stage or even accompanying an entire growth period could give deeper insights into these repeatedly observed findings.

Our detailed analysis of the molecular polymorphism for *Rlm9* in the parents of the mapping population also supports this hypothesis. In particular, non-synonymous amino acid changes, an inserted stop codon and especially the large insertion in *Rlm9* most likely alter the transcript or even interrupt the transcription of the gene in four of the seven parents of the mapping population. Usually, *R* genes encode nucleotide-binding site leucine-rich repeat (NBS-LRR) proteins (McHale et al., 2006). Wall-associated kinase (WAK) and wall-associated kinase-like (WAKL) genes are a newly discovered class of race-specific plant receptor-like kinase resistance genes involved in qualitative resistance (Larkan et al., 2020). However, although some WAKL genes have been shown to be involved in race-specific gene-for-gene interactions, for example *Rlm9* in oilseed rape and *Stb9* in wheat (Keller and Krattinger, 2018), other WAKL genes like *RFO1* (*RESISTANCE TO FUSARIUM OXYSPORUM 1*)/*WAKL22* in Arabidopsis have been found to be involved in broad-spectrum resistance against *F. oxysporum* f. sp. *matthioli* and other races (Diener and Ausubel, 2005) and against *Verticillium longisporum* (Johansson et al., 2006) in Arabidopsis, while *ZmWAK1* confers quantitative resistance to northern corn leaf blight in maize (Hurni et al., 2015). The protein structure of the WAKs and some WAKLs can be divided into an extracellular and an intracellular compartment, which are connected by a transmembrane domain. These proteins are characterized by a cytoplasmic Ser/Thr kinase domain in the cell interior and an extracellular domain that is similar to epidermal growth factor domains in vertebrates (epidermal growth factor; EGF-like domain). This occurs as a calcium-binding EGF domain (EGF-Ca^2+^) and/or as an EGF2-like domain, although in some cases it is slightly degenerate (Verica and He, 2002). The exact function of these EGF-like domains is still largely unclear. However, previous studies demonstrated that EGF-like domains are involved in protein-protein interactions (Kuroda and Tanizawa, 1999). This extracellular domain is disrupted in four of the seven parents in our study by a 6 kb insertion, which might thus be expected to potentially interrupt (unknown) protein-protein-interactions and could consequently impart a quantitative impact on resistance activity. Other parts of the extracellular domain are bound to the pectin of the cell wall (Wagner and Kohorn, 2001), but can also serve as receptors for oligogalacturonides (OGs), which, among other things, arise from mechanical destruction of the pectin and act as DAMPs (damage-associated molecular patterns) to activate the plant immune system (Brutus et al., 2010). This part contains a conserved GUB_WAK domain (galacturonan-binding wall-associated receptor kinase). This suggests that WAKL genes might be involved in more broad-spectrum resistance in some pathogen-host interactions by sensing DAMPs. Hence, our results in oilseed rape suggest a possible dual function of *Rlm9*. On the one hand, *Rlm9* is expected to be involved in race-specific PTI in oilseed rape when challenged with *L. maculans* isolates carrying *AvrLm5-9*, which trigger a strong qualitative gene-for-gene resistance effect in plant genotypes with a functional interacting *Rlm9* gene as in Raman et al. (2018). However, in contrast to Raman et al. (2018), the results in our study suggest that the gene-for-gene interaction for *Rlm9* is not only expressed at the cotyledon stage, but also to a lesser extent in adult plants. In contrast to Raman et al. (2018), we used a *L. maculans* isolate which did not harbor a corresponding avirulence gene (*avrLm5-9*). Thus, even though no gene-for-gene interaction of *Rlm9* with its corresponding avirulence gene is expected in our experiment, still we found a weak expression of quantitative resistance of 5%. This indicates that *Rlm9* may have other additional features triggering quantitative resistance, for example by sensing DAMPs. Only if some functional domains are disrupted, like the EGF-like domain in four of the seven genotypes in our study, might this weak quantitative resistance effect also be lost. All in all, the results of the present study confer with previous studies that observed *R* gene mediated resistance under controlled and field conditions at adult plant stages in *B. napus* (Delourme et al., 2004; Raman et al., 2012). Based on these collective observations, we hypothesize that the role of *Rlm9* in this interaction deserves more detailed functional analysis in future.

## Data Availability Statement

The datasets presented in this study can be found in online repositories. The names of the repository/repositories and accession number(s) can be found below: NCBI (accession: PRJNA751459).

## Author Contributions

CO and RS conceived the idea and sourced the funding. PV, HC, and CO developed the methodology. PV generated the genetic and field data. DA and BK generated the greenhouse data. PV and CO performed data curation. PV, HC, and HL analyzed the sequence and SV data. PV, SW, LE, and IG performed the quantitative genetic analysis. PV, RS, and CO drafted and revised the manuscript. All authors contributed to the article and approved the submitted version.

## Conflict of Interest

The authors declare that the research was conducted in the absence of any commercial or financial relationships that could be construed as a potential conflict of interest.

## Publisher’s Note

All claims expressed in this article are solely those of the authors and do not necessarily represent those of their affiliated organizations, or those of the publisher, the editors and the reviewers. Any product that may be evaluated in this article, or claim that may be made by its manufacturer, is not guaranteed or endorsed by the publisher.

## Footnotes

1
http://population-genetics.uni-giessen.de/~software/

2
https://github.com/groupschoof/AHRD

## Supplementary Material

Supplementary Figure 1 Phenotypic distribution of VDT values from greenhouse screenings for the entire mapping population **(A)** and the individual subfamilies: Adriana × Lorenz **(B)**, Lorenz × Alpaga **(C)**, Lorenz × DK Cabernet **(D)**, Lorenz × Galileo **(E)**, JN × Lorenz **(F)**, and King 10 × Lorenz **(G)**.
