# Supplementary figures and images for: Dissection of Quantitative Blackleg Resistance Reveals Novel Variants of Resistance Gene Rlm9 in Elite Brassica napus
Source: Front Plant Sci. 2021 Nov 18;12:749491. doi: 10.3389/fpls.2021.749491 (PMC8636856; doi:10.3389/fpls.2021.749491)

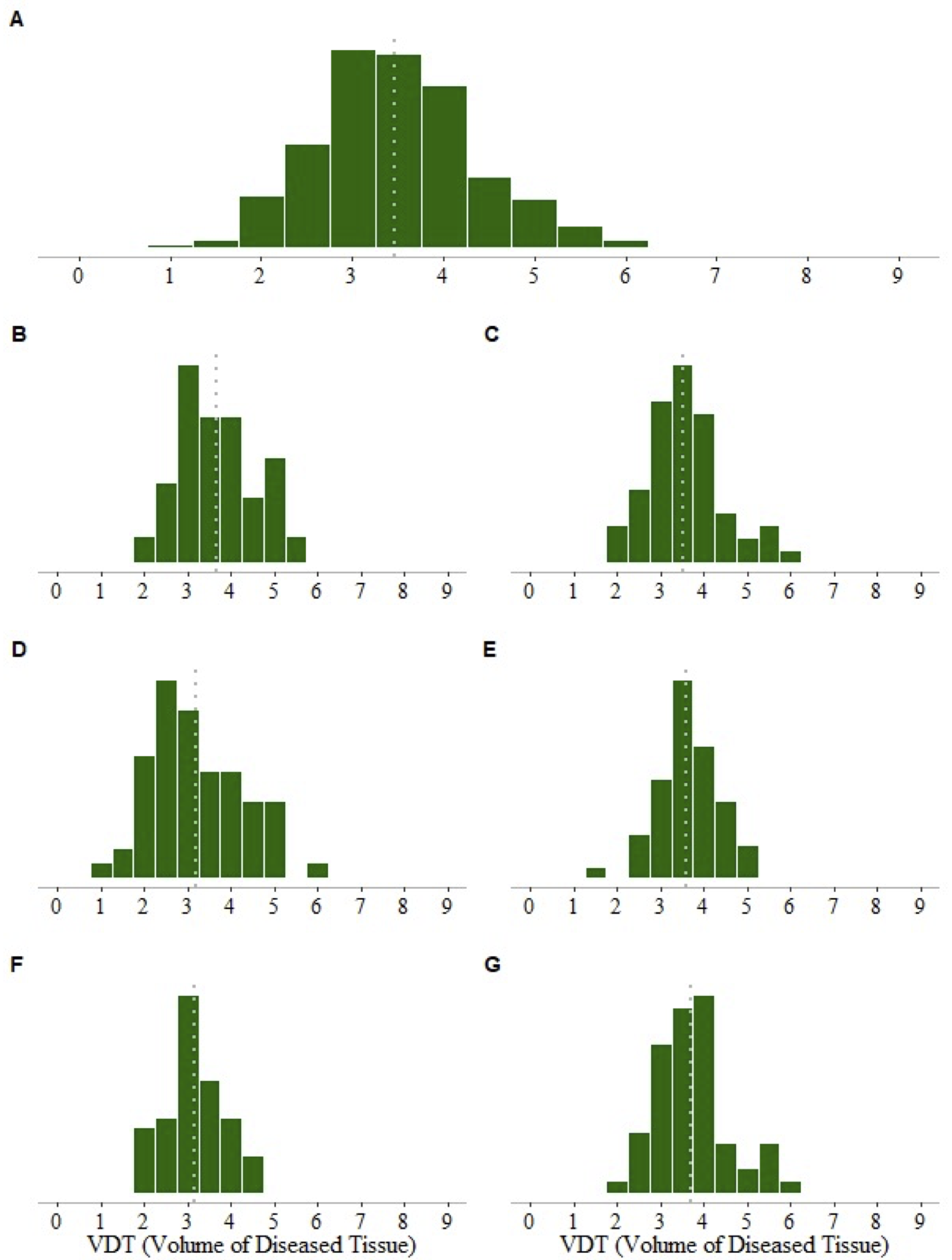

Supplement: Supplementary Figure 1 — Phenotypic distribution of VDT values from greenhouse screenings for the entire mapping population (A) and the individual subfamilies: Adriana × Lorenz (B), Lorenz × Alpaga (C), Lorenz × DK Cabernet (D), Lorenz × Galileo (E), JN × Lorenz (F), and King 10 × Lorenz (G). [file Image_1.TIFF]
